# Resolutions of the Medical Class

**Published:** 1864-01

**Authors:** 


*Resolutions of the Medical Class.*—At a meeting held by the students of "Rush Medical College" for the purpose of considering the death of our fellow student, Mr. R. L. Smith, the following preamble and resolutions were presented and unanimously adopted:

WHEREAS, In the providence of God our young friend and classmate, Mr. R. L. Smith, has been by death removed from our midst, thereby suddenly cutting short, while in the midst of preparatory study, that hope of future usefulness cherished by himself and friends, therefore,

*Resolved*, That while we mourn his loss as a classmate and companion we will cherish his memory with much respect.

*Resolved*, That we tender our deepest sympathy to the family and friends of the deceased in this hour of their affliction.

*Resolved*, That a copy of these resolutions be transmitted to the father of the deceased; and also the "Chicago Medical Journal" for publication.

H. C. HOLLINGSWORTH,

G. B. LESTER,

J. W. THAYER,

J. A. WILLIAMS,

M. JENKINS,

} *Committee.*

